# Differences in intestinal and renal Ca and P uptake in three different breeds of growing-finishing pigs

**DOI:** 10.1080/01652176.2024.2371609

**Published:** 2024-07-04

**Authors:** Chenjian Li, Md. Abul Kalam Azad, Qian Zhu, Yating Cheng, Jue Gui, Bo Song, Zhenlei Zhou, Xiangfeng Kong

**Affiliations:** a CAS Key Laboratory of Agro-ecological Processes in Subtropical Region, Hunan Provincial Key Laboratory of Animal Nutritional Physiology and Metabolic Process, Institute of Subtropical Agriculture, Chinese Academy of Sciences, Changsha, Hunan, China; b College of Veterinary Medicine, Nanjing Agriculture University, Nanjing, Jiangsu, China; c College of Advanced Agricultural Sciences, University of Chinese Academy of Sciences, Beijing, China

**Keywords:** Breed, bone characteristics, calcium, growing-finishing pigs, phosphorus

## Abstract

This study investigated the differences in bone growth and turnover and calcium (Ca) and phosphorus (P) uptake among three different breeds of growing-finishing pigs. Ninety healthy Duroc, Xiangcun black (XCB), and Taoyuan black (TYB) pigs (30 pigs per breed) at 35 day-old (D) with the average body weight (BW) of their respective breed were assigned and raised to 185 D. The results showed that Duroc pigs had higher bone weight and length than the XCB and TYB pigs at 80, 125, and 185 D and the bone index at 185 D (*p* < 0.05). Duroc pigs had higher bone mineral densities (femur and tibia) compared with the other two breeds at 80 D and 125 D, whereas TYB pigs had higher mineral content and bone breaking load (rib) compared with the other two breeds at 185 D (*p* < 0.05). The bone morphogenetic protein-2 and osteocalcin concentrations were higher, and TRACP5b concentration was lower in serum of TYB pigs at 125 D (*p* < 0.05). Meanwhile, 1,25-dihydroxyvitamin D_3_, parathyroid hormone, thyroxine, and fibroblast growth factor 23 concentrations were higher in serum of TYB pigs at 185 D (*p* < 0.05). The TYB pigs had higher apparent total tract digestibility of P at 80 D and 185 D and bone Ca and P contents at 185 D in comparison to the Duroc pigs (*p* < 0.05). Furthermore, gene expressions related to renal uptake of Ca and P differed among the three breeds of pigs. Collectively, Duroc pigs have higher bone growth, whereas TYB pigs have a higher potential for mineral deposition caused by more active Ca uptake.

## 1. Introduction

Bone is the basic body support for mammals. In addition to its conventional functions, such as protection and movement, it is also considered a reservoir of minerals, especially Ca and P (Su et al. 2019). The first 12 weeks of life as well as the growing stage from 10 to 50 kg body weight (BW) are critical for bone formation of pigs which is essential for promoting skeletal development (Tanck et al. 2001). Generally, bone is composed of a collagen matrix with a crystal of filled hydroxyapatite (HAP), and its growth directly affects muscle attachment at the finishing stage of pigs (Garnero 2015). Bone has a coexistence of strength and toughness to satisfy the needs of daily life, benefiting from an optimized constitution of protein and minerals (Micheletti et al. 2022). Bone also plays an important role in the endocrine system. For instance, osteocalcin (OC) secreted by osteoblasts regulates glucose homeostasis after activation (Schwetz et al. 2012). In the first stages of life, bone metabolisms differ when the peak bone mass is achieved or skeletal maturation is completed. At this age, bone synthesis and formation and the longitudinal and transverse growth of bone are the predominant processes to maintain a dynamic balance of bone formation and bone functions (Taylor et al. 2013). However, less information exists about bone growth and its turnover indicators related to development in Chinese domestic pigs. Therefore, bone growth and its turnover synthesis and formation evaluation in Chinese domestic pigs is necessary due to its direct reflection on growing-finishing pigs’ growth behavior and health conditions.

Calcium (Ca) is one of the most abundant minerals in all animals and is involved in several essential life activities, including nerve excitation, blood coagulation, and bone growth. The biological function of Ca depends on the concentration of Ca stored in bones and the remaining turnover Ca in the extracellular fluid. The levels of parathyroid hormone (PTH), vitamin D, and calcitonin (CT) are important indicators for regulating blood Ca concentration, as well as the intestinal absorption, renal reabsorption, and the storage of Ca in bones (Bass and Chan 2006). Phosphorus (P) also plays important roles in intracellular metabolism, enzyme activity, and bone mineralization (Berndt and Kumar 2009). The P balance in pigs also regulates nutritional absorption from diets in the intestine and tubular reabsorption in the kidney (Berndt et al. 2005). The presence of P in the gut-renal axis has been found to regulate intestinal P absorption and renal P excretion (Berndt et al. 2007). P homeostasis leads to an imbalance of P metabolic pathways in the intestine, kidney, and bone (Chowdhury et al. 2008). Moreover, bone absorption and mineralization are critical for regulating Ca and P homeostasis (Renkema et al. 2008). Thus, due to the potential interrelation among Ca, P, and bone, it is necessary to evaluate differences in bone parameters of pigs caused by Ca and P metabolism.

China has a large number of domestic pig breeds with specific characteristics, such as high fertility and good meat quality (Yang et al. 2003). Moreover, Chinese domestic pigs have differences in size, growth, and other characteristics compared with commercial pigs (Gong et al. 2019). Duroc pigs are widely use in genetic improvement as the terminal sire for crossbreeding for their higher average daily gain (ADG) and lean meat characteristics (Choi et al. 2014). As a domestic pig breed of Hunan province in China, Taoyuan black (TYB) pig has its own superiority, including the higher intramuscular fat content and adaptation to roughage feeding (Xu et al. 2015). The Xiangcun black pig (XCB; Duroc × TYB) is a new lean-meat pig breed in China and has gained potential interest in the pork industry (Zhang et al. 2021). To date, changes in bone growth and its turnover synthesis and formation associated with growth behavior and health conditions of growing-finishing pigs and their intestinal absorption and gene expression related to renal Ca and P uptake remain unknown. Therefore, the present study aimed to explore the differences in gene expression related to renal uptake of Ca and P of these pig breeds, which might have great significance in modifying or improving the Ca and P content in domestic pigs’ diets for utilizing and protecting Chinese domestic pig germplasm resources. We hypothesized that XCB and TYB pigs might have differences in bone characteristics and Ca and P metabolism from Duroc pigs, which may provide new information for dietary intervention for domestic pig breeding. To test these hypotheses, we selected Duroc, XCB, and TYB pigs to analyze developmental changes in bone growth, intestinal absorption, and gene expression related to renal uptake of Ca and P at 80 day-old (D), 125 D, and 185 D.

## 2. Material and methods

### 2.1. Animal management and diets

Ninety healthy Duroc, TYB, and XCB pigs from 30 litters (10 litters from each breed and three pigs from each litter) at 35 D with average BW of their respective litter were selected and transferred to single pens until sample collection (one-half of the pigs were female and one-half of the pigs were castrated males). Each pig was housed in a single pen (0.70 × 1.50 m), and different pig breeds were kept separately in one barn. Feeding and housing management for all pigs were maintained consistent conditions according to standard management of the commercial pig farm. Duroc pigs were purchased from Tianxin Breeding Co., Ltd. (Changsha, China), and XCB and TYB pigs were purchased from Xiangcun High-Tech Agricultural Co., Ltd. (Loudi, China). All experimental animals were fed pre-nursery and late-nursery diets from 35 to 80 D, growing diets from 81 to 125 D, and finishing diets from 126 to 185 D. Animal housing pens were provided with a single-hole feeder and water nipples to access feed and water *ad libitum* at all time. The composition and nutrient levels of the experimental diets (Table S1) for three different breeds of pigs met the Chinese local swine nutrient requirements (GB NY/T 65 2004), and the premixes met the National Research Council (NRC 2012) diet requirements. Experimental pigs were housed in a well-ventilated room with a constant temperature (23–26 °C) and humidity (60 ± 5%).

### 2.2. Sample collection

Within one week before 80, 125, and 185 D, 0.1% TiO_2_ was added to the diets as an indicator, and the fecal (300 g from 10 pigs/breed) and diet samples were collected and dried completely in an oven at 75 °C to determine the apparent total tract digestibility (ATTD). At 80, 125, and 185 D, a total of 30 pigs (10 pigs/breed) at each age stage were weighed after overnight fasting to evaluate the ADG, average daily feed intake (ADFI), and feed/gain ratio (F:G) during 35–80 D, 80–125 D, and 125–185 D of three different breeds of pigs. Blood samples (10 mL) were collected from 10 pigs per breed at each age stage (90 pigs for three age stages) to obtain serum by centrifuging at 4 °C and 3500×*g* for 15 min and then stored at −80 °C for further determination of Ca and P concentrations and biochemical indicators related to Ca and P metabolism. According to commercial procedures, all pigs were euthanized by electrical stunning (120 V, 200 Hz) and then dissected for sampling. The duodenum (posterior section), jejunum (10 cm below the duodenum-jejunum junction), ileum (10 cm above the ileocecal junction), and kidney (middle portion of the kidney; half of the renal cortex and half of the medulla) were separated successively, and the intestinal contents were washed with normal saline. Intestinal and renal samples were collected and immediately flash-frozen in liquid nitrogen, and then stored at −80 °C for further analysis of gene expressions. The femur and tibia of the left hind limb, left fourth rib, and fourth lumbar vertebrae of each pig were collected (10 samples per pig breed at each stage) after removing all attached tissues. Lumbar vertebrae samples were not collected at 185 D because those were cut in half during harvest. All bone samples were stored at −20 °C for further analysis of bone parameters.

### 2.3. Measurement of bone parameters

Bone samples were thawed thoroughly, and moisture from the bone surface was removed with absorbent paper. The weight and length of all bones were separately measured using the precision electrical balance (Yao Xin Electronic Technology Co. Ltd., Shanghai, China) and Vernier caliper (Heng Liang Technology Co. Ltd., Zhengzhou, China). The bone index was calculated according to the following formula: bone index (g/kg) = bone weight (g)/BW (kg) as described previously (Zhou et al. 2009). Bone breaking load of the femur (left hind limb), tibia (left hind limb), and ribs (left fourth) was measured by a three-point bending test using a universal material machine (LR10K Plus, Lloyd Instruments Co. Ltd., West Sussex, UK) as described previously by Jämsä et al. (1998). Briefly, the position of the midpoint of each bone was recorded using a Vernier caliper (Heng Liang Technology Co. Ltd., Zhengzhou, China) before measuring the bone breaking load. The relevant parameters of the instrument were set as follows: preload 5.6 N and preload velocity 21 mm/min. The test was stopped after the stress curve reached the upper yield point. The compression curves were analyzed using the NEXYGEN PLUS software, and then bone breaking load values were recorded.

Bone mineral density (BMD) and bone mineral content (BMC) were determined using a Dual X-ray Absorptiometry (InAlyzer, Medikors, Inc., Gyeonggi-do, Korea) as described previously (Wang et al. 2021). After calibrating with a standard mold, all bone samples were placed in the testing area in the same position. The high and low energy parameters were 80 kVp/1.0 mA and 55 kVp/1.25 mA, respectively. After the test, the images were saved, and the analyzed area was selected to determine the BMD and BMC.

### 2.4. Measurement of bone ash, Ca, and P contents

Approximately 0.20 g of the femur, tibia, and rib samples (middle section of each bone) were dried in an oven at 70 °C for 12 h and then degreased in petroleum ether for 36 h. After air drying for another 12 h, defatted bone samples were weighed and placed in ceramic crucibles. Empty crucibles were weighed in advance to calculate the weight of the samples. Finally, all samples were completely charred in an electric furnace, transferred into a muffle furnace, and ashed at 550 °C for 3.5 h for bone ash measurements. The ash content was calculated as a percentage of the dry weight of the defatted bone.

Ash samples were completely dissolved in HNO_3_ to determine Ca and P contents in bone. The contents of Ca and P in bone were measured (AOAC Int 2019; method 985.01 A, B, and C) using inductively coupled plasma spectroscopy (5110 ICP–OES, Agilent, Santa Clara, CA, USA). The contents of Ca and P in bone were calculated as follows:

$$N_{c}\;(\%)=\frac{N_{w}}{B_{w}}\times 100$$

Where, N_C_ is the content of Ca or P in bone; N_w_ is the weight of Ca or P in bone; B_w_ is the weight of defatted bone.

### 2.5. Measurement of apparent total tract digestibility of Ca and P

Ca and P contents in fecal and feed samples were determined according to the AOAC methods (AOAC Int 2019; method 985.01 A, B, and C) by inductively coupled plasma spectroscopy (5110 ICP–OES, Agilent, Santa Clara, CA, USA). Approximately 0.50 g of fecal or feed samples were transferred into conical flasks to determine TiO_2_. A 10-mL acid mixture (nitric acid: perchloric acid = 9:1) was added into each flask. The flasks were heated at 50 °C for 30 min and then re-heated at 100 °C until no red-brown smoke was visible. After that, the flasks were heated at 180 °C for 0.5 h and 280 °C for 1 h, respectively, until the remaining volume of solutions inside the flask was less than 1 mL. After cooling at room temperature, 2 g ammonium sulfate and 5 mL sulfuric acid were added to each flask. The flasks were then heated at 300 °C for 40 min, 350 °C for 10 min, and 370 °C for 20 min. Finally, the samples were transferred into 50 mL volumetric flasks and measured Ti concentration dissolved in fecal and feed samples by ICP–OES. The standard solution concentrations for Ti were 1, 2, 5, 10, 20, and 50 ppm.

The ATTD of Ca and P was calculated according to the following formula:

$$D_{N}(\%)=(1-\frac{I_{R}}{I_{F}}\times \frac{N_{F}}{N_{R}})\times 100$$

Where, D_N_ is the ATTD coefficient of Ca or P; I*_R_* is the Ti content in feed; I*_F_* is the Ti content in feces; N*_F_* is the Ca or P content in feces (% DM); N*_R_* is the Ca or P content in diet (% DM).

### 2.6. Measurement of serum Ca and P concentrations

The serum Ca and P concentrations were evaluated by biochemical kits purchased from the Leadman Biochem. Tech. Co., Ltd. (Beijing, China), using an automatic biochemical analyzer (Cobas c311, F. Hoffman-La Roche Ltd., Basel, Switzerland) following the manufacturer’s protocols.

### 2.7. Measurement of serum biochemical indicators

The bone morphogenetic protein-2 (BMP-2; HY-10319P1), bone alkaline phosphatase (BALP; HY-10324P1), OC (HY-01309P1), procollagen I N-terminal propeptide (PINP; HY-10327P1), tartrate-resistant acid phosphatase 5b (TRACP5b; HY-10320P1), type I collagen carboxy-terminal peptide (CTX-1; HY-10070P1), CT (HY-10293P1), 1,25-dihydroxyvitamin D_3_ (HY-10346P1), PTH (HY-10286P1), thyroxine (T4; HY-10315P1), and fibroblast growth factor 23 (FGF23; HY-10299P1) in serum were measured using commercially available enzyme-linked immunosorbent assay kits (Shanghai Huyu Biotechnology Co., Ltd., Shanghai, China) and Microplate Reader (Infinite M200 PRO, Tecan, Switzerland) following the manufacturer’s protocols.

### 2.8. Intestinal and renal RNA extraction and gene expression analysis

The AG RNAex Pro reagent (Accurate Biology, Changsha, China) was used to extract the total RNA from the duodenum, jejunum, ileum, and kidney according to the manufacturer’s guidelines. A NanoDrop ND-2000 spectrophotometer (Thermo Fisher Scientific, Waltham, MA, USA) and agarose gel electrophoresis were used to determine the concentration and purity of the extracted RNA. To obtain cDNA for quantitative PCR analysis, the total RNA (1000 ng) was reversely transcribed using the Evo M-MLV RT Kit with gDNA Clean. The Light Cycler^®^ 480II Real-Time PCR System (Roche, Basel, Switzerland) with SYBR^®^ Green Premix Pro Taq HS qPCR Kit (Accurate Biology) were used for RT-PCR analysis. Specific primers (Table S2) of the target genes were designed and synthesized by Sangon Biotech Co., Ltd. (Shanghai, China). A 10-μL reaction system was used for RT-PCR analysis, contained 0.25 μL of each primer (10 μM), 5.0 μL 2 × SYBR^®^ Green Pro Taq HS Premix, 0.3 μL cDNA, and 4.2 μL RNase free water. The PCR cycling conditions were set as follows: 95 °C for 30 s for initial denaturation, followed by 45 circles of denaturation at 95 °C for 5 s and annealing at 60 °C for 30 s, and a final extension at 72 °C for 30 s. The 2^−△△Ct^ method (Schmittgen and Livak 2008) was used to calculate the levels of gene expression.

### 2.9. Statistical analysis

All data were analyzed using the SPSS statistical software 23.0 (IBM Corp., Chicago, IL, USA). The comparative analysis among different groups was performed by one-way analysis of variance (ANOVA, LSD, and Tamhane T2). All data are expressed as means with their standard errors of the means (SEM) and *P* values. The individual pens were considered the experimental unit for the growth performance, bone parameters, ash, Ca, and P contents analyses, and individual pigs were considered the experimental unit for gene expression related to Ca and P uptake analyses. Statistical significant differences among different breed of pigs were considered when *p* < 0.05.

## 3. Results

### 3.1. Differences in body weight and feed intake among three breeds of growing-finishing pigs

The XCB and TYB pigs had lower (*p* < 0.001) BW, ADG, and ADFI during 35–80 D, 80–125 D, and 125–185 D compared to the Duroc pigs. Moreover, XCB pigs had higher (*p* < 0.01) F:G in comparison to the Duroc and TYB pigs during 35–80 D (Cheng et al. 2023; Table S3).

### 3.2. Differences in bone parameters among three breeds of growing-finishing pigs

The XCB and TYB pigs had shorter and lighter (*p* < 0.001) femur, tibia, rib, and lumbar vertebrae in comparison to the Duroc pigs regardless of age (lumbar vertebrae were not collected at 185 D; Tables 1 and 2). The TYB pigs had longer femur and heavier femur, tibia, and rib at 185 D, as well as heavier femur and tibia at 80 D than the XCB pigs (*p* < 0.001). Regardless of pig breed, the length and weight of the femur, tibia, and rib were increased (*p* < 0.001) with the age of pigs (Tables 1 and 2).

**Table 1.** Differences in bone length (cm) among three breeds of growing-finishing pigs.

Item	Duroc pig	XCB pig	TYB pig	SEM	*P*-values
Femur					
80 D	126.77^Ca^	98.42^Cb^	99.10^Cb^	2.79	<0.001
125 D	168.20^Ba^	133.67^Bb^	134.15^Bb^	3.53	<0.001
185 D	204.31^Aa^	167.34^Ac^	178.40^Ab^	3.24	<0.001
SEM	7.07	5.57	6.84		
*P*-values	<0.001	<0.001	<0.001		
Tibia					
80 D	115.82^Ca^	91.74^Cb^	95.25^Cb^	2.37	<0.001
125 D	157.23^Ba^	124.83^Bb^	125.36^Bb^	3.46	<0.001
185 D	183.83^Aa^	154.84^Ab^	160.27^Ab^	2.67	<0.001
SEM	6.29	5.17	5.70		
*P*-values	<0.001	<0.001	<0.001		
Rib					
80 D	114.34^Ca^	93.34^Cb^	94.19^Cb^	2.17	<0.001
125 D	152.37^Ba^	125.91^Bb^	126.85^Bb^	2.79	<0.001
185 D	174.86^Aa^	153.19^Ab^	159.60^Ab^	2.26	<0.001
SEM	5.72	4.83	5.67		
*P*-values	<0.001	<0.001	<0.001		
Lumbar vertebrae					
80 D	24.68^Ba^	18.38^Bb^	18.93^Bb^	0.60	<0.001
125 D	32.68^Aa^	26.58^Ab^	26.84^Ab^	0.66	<0.001
185 D*	—	—	—		
SEM	1.04	1.04	1.10		
*P*-values	<0.001	<0.001	<0.001		

Data are presented as means, SEM, and *P*-values. The replicates at 80, 125, and 185 D were 10 pigs per breed. ^a–c^The mean values with different small superscripts in the same row indicate significant differences among different pig breeds (*p* < 0.05). ^A–C^The mean values with different capital superscripts in the same column indicate significant differences among different day-old (*p* < 0.05). XCB, Xiangcun black; TYB, Taoyuan black; 80 D, 80 day-old; 125 D, 125 day-old; 185 D, 185 day-old.

*Lumbar vertebrae samples were not collected at 185 D due to those were cut in half during harvest.

**Table 2.** Differences in bone weight (g) among different breeds of growing-finishing pigs.

Item	Duroc pig	XCB pig	TYB pig	SEM	*P*-values
Femur					
80 D	103.62^Ca^	44.03^Cc^	53.07^Cb^	5.47	<0.001
125 D	236.64^Ba^	110.11^Bb^	118.88^Bb^	12.09	<0.001
185 D	365.19^Aa^	202.68^Ac^	236.19^Ab^	12.09	<0.001
SEM	23.83	13.07	16.43		
*P*-values	<0.001	<0.001	<0.001		
Tibia					
80 D	66.37^Ca^	29.92^Cc^	35.42^Cb^	3.39	<0.001
125 D	145.98^Ba^	71.90^Bb^	75.76^Bb^	7.20	<0.001
185 D	221.14^Aa^	122.86^Ac^	144.90^Ab^	8.67	<0.001
SEM	14.14	7.62	9.76		
*P*-values	<0.001	<0.001	<0.001		
Rib					
80 D	10.66^Ca^	4.20^Cb^	5.14^Cb^	0.62	<0.001
125 D	28.09^Ba^	10.92^Bb^	13.10^Bb^	1.63	<0.001
185 D	44.28^Aa^	22.46^Ac^	31.29^Ab^	2.05	<0.001
SEM	3.19	1.56	2.36		
*P*-values	<0.001	<0.001	<0.001		
Lumbar vertebrae					
80 D	23.64^Ba^	11.21^Bb^	12.75^Bb^	1.21	<0.001
125 D	53.52^Aa^	29.28^Ab^	31.15^Ab^	2.46	<0.001
185 D*	—	—	—		
SEM	3.86	2.35	2.66		
* P*-values	<0.001	<0.001	<0.001		

Data are presented as means, SEM, and *P*-values. The replicates at 80, 125, and 185 D were 10 pigs per breed. ^a–c^The mean values with different small superscripts in the same row indicate significant differences among different pig breeds (*p* < 0.05). ^A–C^The mean values with different capital superscripts in the same column indicate significant differences among different day-old (*p* < 0.05). XCB, Xiangcun black; TYB, Taoyuan black; 80 D, 80 day-old; 125 D, 125 day-old; 185 D, 185 day-old.

*Lumbar vertebrae samples were not collected at 185 D due to those were cut in half during harvest.

The rib index of XCB and TYB pigs was lower (*p* < 0.001) at 125 D, as well as the femur and tibia indexes of XCB and TYB pigs and the rib index of XCB pigs at 185 D than the Duroc pigs (Table 3). The rib index of TYB pigs was higher (*p* < 0.001) at 185 D than the XCB pigs. At 125 D, the femur index of Duroc pigs was lower (*p* < 0.05) but it was higher (*p* < 0.001) in the XCB and TYB pigs than the pigs at 80 D. The tibia and rib indexes of XCB pigs (*p* < 0.001) and the tibia index of TYB pigs (*p* < 0.001) were increased at 125 D, while the lumbar vertebrae index of Duroc (*p* < 0.05), XCB (*p* < 0.001), and TYB (*p* < 0.05) pigs was decreased at 125 D, when compared with the pigs at 80 D. Compared to 125 D, the femur and tibia indexes were increased (*p* < 0.01) in the Duroc pigs and decreased (*p* < 0.001) in the XCB pigs at 185 D; moreover, the rib index was increased (*p* < 0.05) in the Duroc pigs at 185 D (Table 3).

**Table 3.** Differences in bone index (g/kg) among different breeds of growing-finishing pigs.

Item	Duroc pig	XCB pig	TYB pig	SEM	*P*-values
Femur					
80 D	4.55^B^	4.69^A^	4.71^A^	0.10	0.779
125 D	3.86^C^	3.11^B^	3.18^B^	0.14	0.057
185 D	5.19^Aa^	2.80^Cb^	3.09^Bb^	0.23	<0.001
SEM	0.16	0.17	0.20		
*P*-values	0.002	<0.001	<0.001		
Tibia					
80 D	2.91^AB^	3.19^A^	3.14^A^	0.07	0.225
125 D	2.38^B^	2.03^B^	2.01^B^	0.08	0.127
185 D	3.14^Aa^	1.70^Cb^	1.89^Bb^	0.13	<0.001
SEM	0.09	0.13	0.14		
*P*-values	0.003	<0.001	<0.001		
Rib					
80 D	0.47^B^	0.45^A^	0.46	0.02	0.874
125 D	0.46^Ba^	0.31^Bb^	0.35^b^	0.02	0.007
185 D	0.63^Aa^	0.31^Bc^	0.41^b^	0.03	<0.001
SEM	0.03	0.02	0.02		
*P*-values	0.003	<0.001	0.075		
Lumbar vertebrae					
80 D	1.04^A^	1.19^A^	1.12^A^	0.03	0.108
125 D	0.87^B^	0.83^B^	0.83^B^	0.03	0.854
185 D*	—	—	—		
SEM	0.04	0.05	0.07		
*P*-values	0.036	<0.001	0.021		

Data are presented as means, SEM, and *P*-values (*n* = 10). The replicates at 80, 125, and 185 D were 10 pigs per breed. ^a–c^The mean values with different small superscripts in the same row indicate significant differences among different pig breeds (*p* < 0.05). ^A–C^The mean values with different capital superscripts in the same column indicate significant differences among different day-old (*p* < 0.05). XCB, Xiangcun black; TYB, Taoyuan black; 80 D, 80 day-old; 125 D, 125 day-old; 185 D, 185 day-old.

*Lumbar vertebrae samples were not collected at 185 D due to those were cut in half during harvest.

### 3.3. Differences in bone mineral density and contents among three breeds of growing-finishing pigs

The XCB and TYB pigs had lower femur density (*p* < 0.05) at 125 D and the tibia density (*p* < 0.01) at 80 D and 125 D, as well as the lumbar vertebrae density (*p* < 0.05) at 125 D, and the TYB pigs had lower rib density (*p* < 0.05) at 80 D than the Duroc pigs (Table 4). The femur, tibia, rib, and lumbar vertebrae densities of all breeds of pigs were greater (*p* < 0.001) at 125 D than the pigs at 80 D. Moreover, the femur, tibia, and rib densities of all breeds of pigs were increased (*p* < 0.001) at 185 D compared to the pigs at 125 D.

**Table 4.** Differences in bone mineral density (g/cm^3^) among different breeds of growing-finishing pigs.

Item	Duroc pig	XCB pig	TYB pig	SEM	*P*-values
Femur					
80 D	0.34^Ca^	0.29^Cb^	0.28^Cb^	0.01	0.010
125 D	0.69^Ba^	0.55^Bb^	0.58^Bb^	0.19	0.006
185 D	1.04^A^	1.01^A^	0.97^A^	0.03	0.650
SEM	0.07	0.06	0.06		
*P*-values	<0.001	<0.001	<0.001		
Tibia					
80 D	0.32^Ca^	0.27^Cb^	0.26^Cb^	0.01	0.002
125 D	0.64^Ba^	0.49^Bb^	0.50^Bb^	0.02	0.001
185 D	0.98^A^	0.90^A^	0.84^A^	0.03	0.194
SEM	0.06	0.05	0.05		
*P*-values	<0.001	<0.001	<0.001		
Rib					
80 D	0.13^Ca^	0.11^Cab^	0.10^Cb^	0.00	0.038
125 D	0.18^B^	0.17^B^	0.17^B^	0.00	0.730
185 D	0.29^A^	0.34^A^	0.35^A^	0.01	0.133
SEM	0.02	0.02	0.02		
*P*-values	<0.001	<0.001	<0.001		
Lumbar vertebrae					
80 D	0.20^B^	0.19^B^	0.18^B^	0.00	0.259
125 D	0.37^Aa^	0.33^Ab^	0.32^Ab^	0.01	0.028
185 D*	—	—	—		
SEM	0.02	0.02	0.02		
*P*-values	<0.001	<0.001	<0.001		

Data are presented as means, SEM, and *P*-values. The replicates at 80, 125, and 185 D were 10 pigs per breed. ^a–c^The mean values with different small superscripts in the same row indicate significant differences among different pig breeds (*p* < 0.05). ^A–C^The mean values with different capital superscripts in the same column indicate significant differences among different day-old (*p* < 0.05). XCB, Xiangcun black; TYB, Taoyuan black; 80 D, 80 day-old; 125 D, 125 day-old; 185 D, 185 day-old.

*Lumbar vertebrae samples were not collected at 185 D due to those were cut in half during harvest.

The mineral contents in the femur, tibia, rib, and lumbar vertebrae were lower (*p* < 0.001) in the XCB and TYB pigs at 80 D and 125 D, as well as in the femur and tibia at 185 D than the Duroc pigs (Table 5). Compared to the Duroc and TYB pigs, the mineral content in the rib was lower (*p* < 0.001) in the XCB pigs at 185 D. Mineral contents in the femur, tibia, rib, and lumbar vertebrae of all breeds of pigs were higher (*p* < 0.001) at 125 D than the pigs at 80 D. Moreover, mineral contents in the femur, tibia, and rib of all pigs were higher (*p* < 0.001) at 185 D than in the pigs at 125 D.

**Table 5.** Differences in bone mineral content (g) among different breeds of growing-finishing pigs.

Item	Duroc pig	XCB pig	TYB pig	SEM	*P*-values
Femur					
80 D	14.77^Ca^	6.96^Cb^	7.83^Cb^	0.80	<0.001
125 D	52.62^Ba^	24.70^Bb^	27.47^Bb^	2.75	<0.001
185 D	106.65^Aa^	68.76^Ab^	73.39^Ab^	4.20	<0.001
SEM	8.86	5.29	5.83		
*P*-values	<0.001	<0.001	<0.001		
Tibia					
80 D	10.78^Ca^	5.39^Cb^	5.83^Cb^	0.58	<0.001
125 D	36.80^Ba^	17.23^Bb^	17.88^Bb^	1.98	<0.001
185 D	75.35^Aa^	45.70^Ab^	48.18^Ab^	3.20	<0.001
SEM	6.21	3.47	3.84		
*P*-values	<0.001	<0.001	<0.001		
Rib					
80 D	1.48^Ca^	0.74^Cb^	0.86^Cb^	0.08	<0.001
125 D	4.54^Ba^	2.08^Bb^	2.45^Bb^	0.24	<0.001
185 D	8.45^Aa^	5.97^Ab^	8.26^Aa^	0.35	0.001
SEM	0.66	0.45	0.70		
*P*-values	<0.001	<0.001	<0.001		
Lumbar vertebrae					
80 D	2.74^Ba^	1.53^Bb^	1.63^Bb^	0.13	<0.001
125 D	8.90^Aa^	4.98^Ab^	5.20^Ab^	0.41	<0.001
185 D*	—	—	—		
SEM	0.83	0.46	0.49		
*P*-values	<0.001	<0.001	<0.001		

Data are presented as means, SEM, and *P*-values. The replicates at 80, 125, and 185 D were 10 pigs per breed. ^a–c^The mean values with different small superscripts in the same row indicate significant differences among different pig breeds (*p* < 0.05). ^A–C^The mean values with different capital superscripts in the same column indicate significant differences among different day-old (*p* < 0.05). XCB, Xiangcun black; TYB, Taoyuan black; 80 D, 80 day-old; 125 D, 125 day-old; 185 D, 185 day-old.

*Lumbar vertebrae samples were not collected at 185 D due to those were cut in half during harvest.

### 3.4. Differences in bone breaking load and ash content among three breeds of growing-finishing pigs

The bone breaking load of the femur and tibia of XCB and TYB pigs were lower (*p* < 0.001) in comparison to the Duroc pigs, regardless of age (Table 6). The rib breaking load of XCB and TYB pigs was lower (*p* < 0.001) at 125 D than the Duroc pigs. Compared to Duroc and XCB pigs, the rib breaking load of TYB pigs was higher (*p* < 0.001) at 185 D. The femur and tibia breaking load of Duroc, XCB, and TYB pigs and rib breaking load of Duroc and XCB pigs at 125 D were higher (*p* < 0.001) than those pigs at 80 D. Moreover, the femur, tibia, and rib breaking load of Duroc, XCB, and TYB pigs at 185 D were higher (*p* < 0.05) than those pigs at 125 D.

**Table 6.** Differences in bone breaking load (N) among different breeds of growing-finishing pigs.

Item	Duroc pig	XCB pig	TYB pig	SEM	*P*-values
Femur					
80 D	716.21^Ca^	484.19^Cb^	456.24^Cb^	31.82	<0.001
125 D	1716.98^Ba^	1035.17^Bb^	1045.39^Bb^	75.58	<0.001
185 D	2534.35^Aa^	1891.76^Ab^	2043.80^Ab^	83.86	0.001
SEM	170.13	117.46	146.33		
*P*-values	<0.001	<0.001	<0.001		
Tibia					
80 D	803.28^Ca^	467.32^Cb^	441.36^Cb^	38.82	<0.001
125 D	1922.00^Ba^	1042.04^Bb^	1057.52^Bb^	91.55	<0.001
185 D	2923.23^Aa^	1936.83^Ab^	2089.60^Ab^	121.86	<0.001
SEM	200.05	125.25	151.82		
*P*-values	<0.001	<0.001	<0.001		
Rib					
80 D	39.59^C^	30.33^C^	38.94^B^	1.88	0.073
125 D	93.63^Ba^	62.81^Bb^	54.89^Bb^	4.61	0.001
185 D	136.18^Ab^	118.89^Ab^	214.40^Aa^	12.13	0.002
SEM	9.74	7.83	18.45		
*P*-values	<0.001	<0.001	<0.001		

Data are presented as means, SEM, and *P*-values. The replicates at 80, 125, and 185 D were 10 pigs per breed. ^a–c^The mean values with different small superscripts in the same row indicate significant differences among different pig breeds (*p* < 0.05). ^A–C^The mean values with different capital superscripts in the same column indicate significant differences among different day-old (*p* < 0.05). XCB, Xiangcun black; TYB, Taoyuan black; 80 D, 80 day-old; 125 D, 125 day-old; 185 D, 185 day-old.

**Figure 1.**
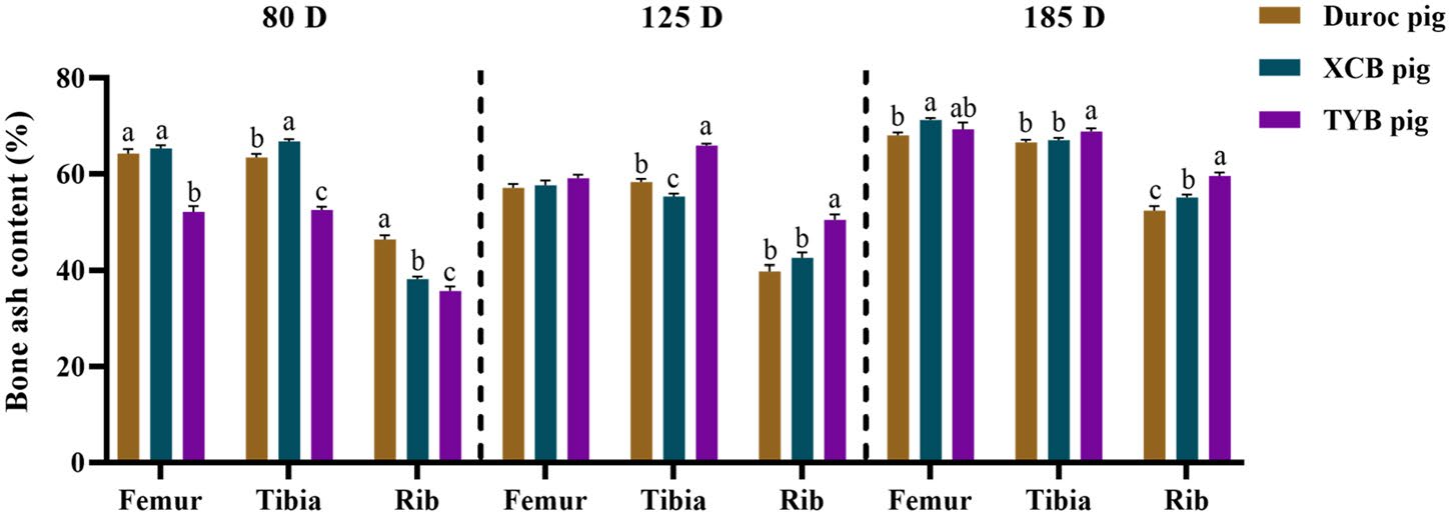
Differences in bone ash content among three breeds of growing-finishing pigs. Values are shown as means with their SEM. The replicates at 80, 125, and 185 D were six pigs per breed. Different small letters (a, b, and c) indicate significant differences among different pig breeds at the same day-old (p < 0.05). 80, 125, and 185 D, represent 80, 125, and 185 day-old, respectively.

As shown in Figure 1, the ash content was lower (*p* < 0.05) in the femur, tibia, and rib of TYB pigs at 80 D, whereas it was higher (*p* < 0.05) in the tibia and rib of TYB pigs at 125 and 185 D, when compared with the Duroc and XCB pigs. Compared with the Duroc pigs, the ash content was higher in the tibia of XCB pigs at 80 D but it was lower at 125 D (*p* < 0.05). The ash content was lower in the rib at 80 D but higher at 185 D, while it was higher in the femur at 185 D in the XCB pigs in relation to the Duroc pigs (*p* < 0.05).

### 3.5. Differences in bone metabolism marker levels among three breeds of growing-finishing pigs

**Figure 2.**
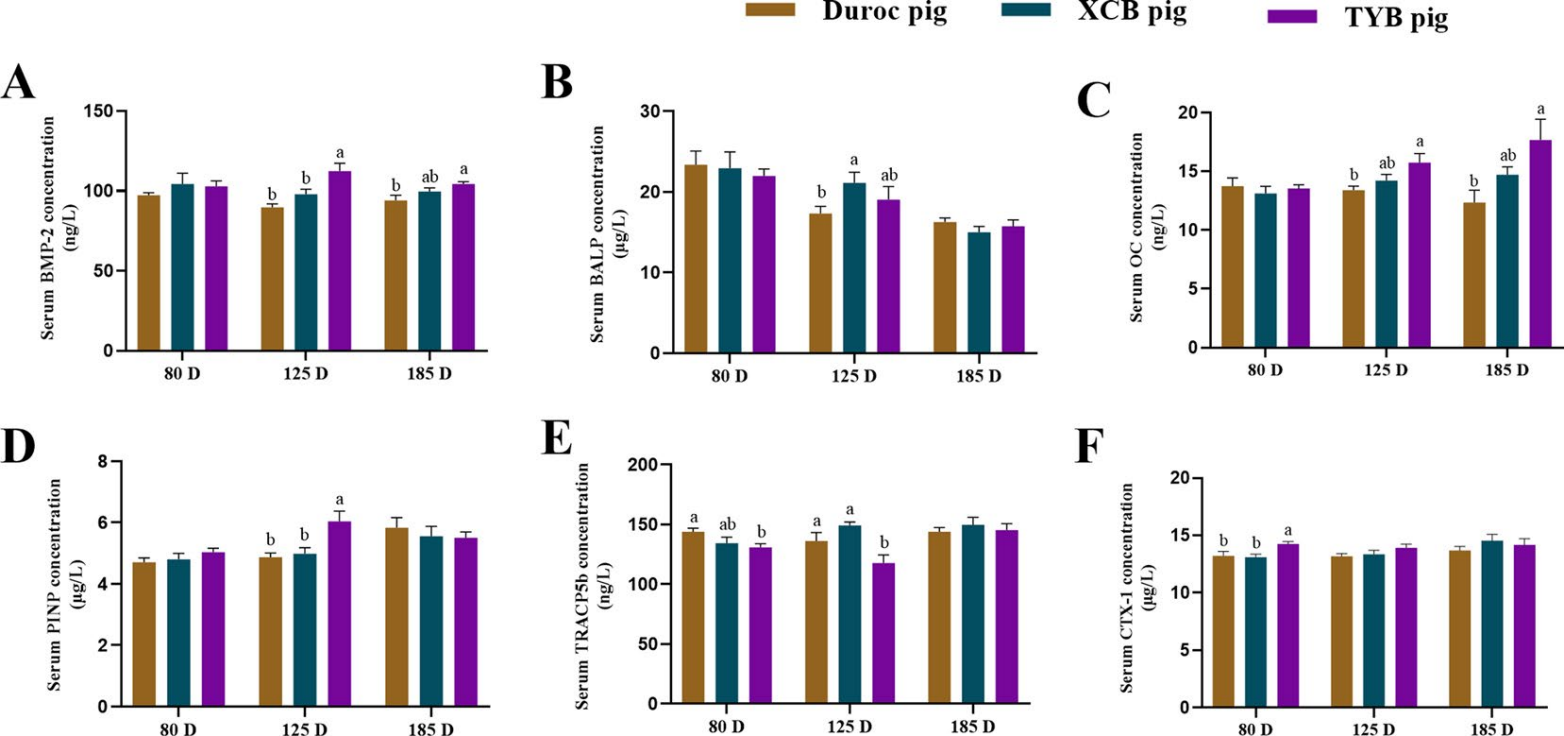
Differences in serum concentrations of osteogenesis factors (A–D) and bone resorption markers (E–F) among three breeds of growing-finishing pigs. Different small letters (a and b) indicate significant differences among different pig breeds at the same day-old (p < 0.05). 80, 125, and 185 D, represent 80, 125, and 185 day-old, respectively. BMP-2 = bone morphogenetic protein-2; BALP = bone alkaline phosphatase; OC = osteocalcin; PINP = procollagen I N-terminal propeptide; TRACP 5b = tartrate-resistant acid phosphatase 5b; CTX-1 = type I collagen carboxy-terminal peptide. The replicates of Duroc, XCB, and TYB pigs at 80 D were 9, 9, and 10, respectively. The replicates of Duroc, XCB, and TYB pigs at 125 D were 9, 9, and 8, respectively. The replicates of Duroc, XCB, and TYB pigs at 185 D were 9, 10, and 9, respectively.

The serum concentrations of BMP-2, OC, and PINP of TYB pigs were higher (*p* < 0.05) at 125 D, as well as BMP-2 and OC at 185 D, while serum BALP of XCB pigs was higher (*p* < 0.05) at 125 D compared with the Duroc pigs (Figure 2). The serum concentrations of BMP-2 and PINP of XCB pigs were lower (*p* < 0.05) at 125 D than the TYB pigs (Figure 2A–D). The serum TRACP5b concentration was higher (*p* < 0.05) in the Duroc pigs at 80 D and 125 D, as well as in the XCB pigs at 125 D, when compared with the TYB pigs. The serum CTX-1 concentration of Duroc and XCB pigs was lower (*p* < 0.05) at 80 D than the TYB pigs (Figure 2E–F).

### 3.6. Differences in apparent total tract digestibility of Ca and P among three breeds of growing-finishing pigs

The Ca ATTD of Duroc and TYB pigs was higher (*p* < 0.01) than the XCB pigs at 80 D. The P ATTD of TYB pigs was higher (*p* < 0.05) at 80 D, as well as that of XCB and TYB pigs at 185 D in comparison with the Duroc pigs (Table 7). The Ca ATTD of Duroc (*p* < 0.001) and XCB (*p* < 0.05) pigs was higher, while the P ATTD of Duroc (*p* < 0.001), XCB (*p* < 0.01), and TYB (*p* < 0.001) pigs was lower at 125 D, when compared with those pigs at 80 D. Moreover, the Ca ATTD of all pig breeds was lower, as well as the P ATTD of Duroc and TYB pigs at 185 D, when compared with those pigs at 125 D (*p* < 0.01).

**Table 7.** Differences in the ATTD (%) of Ca and P among different breeds of growing-finishing pigs.

Item	Duroc pig	XCB pig	TYB pig	SEM	*P*-values
Ca					
80 D	53.55^Ba^	39.51^Bb^	52.20^Aa^	1.90	0.001
125 D	59.92^A^	55.92^A^	57.17^A^	1.53	0.561
185 D	48.83^C^	44.63^B^	46.60^B^	0.87	0.144
SEM	1.22	2.14	1.38		
*P*-values	<0.001	0.003	0.002		
P					
80 D	47.26^Ab^	47.02^Ab^	54.80^Aa^	1.20	0.013
125 D	42.00^B^	38.08^B^	41.18^B^	1.42	0.501
185 D	29.78^Cb^	34.19^Ba^	33.66^Ca^	0.79	0.039
SEM	1.68	1.70	1.94		
*P*-values	<0.001	0.002	<0.001		

Data are presented as means, SEM, and *P*-values. The replicates at 80, 125, and 185 D were 10 pigs per breed. ^a–c^The mean values with different small superscripts in the same row indicate significant differences among different pig breeds (*p* < 0.05). ^A–C^The mean values with different capital superscripts in the same column indicate significant differences among different day-old (*p* < 0.05). XCB, Xiangcun black; TYB, Taoyuan black; 80 D, 80 day-old; 125 D, 125 day-old; 185 D, 185 day-old.

### 3.7. Differences in serum Ca and P concentrations among three breeds of growing-finishing pigs

Serum P concentration was lower in the TYB pigs (*p* < 0.05) at 80 D, as well as in the XCB pigs (*p* < 0.01) at 185 D, when compared with the Duroc pigs (Table 8). Serum Ca concentration was higher (*p* < 0.05) in the Duroc pigs, whereas serum P concentration was lower (*p* < 0.05) in the TYB pigs at 125 D than the pigs at 80 D. In addition, serum P concentration was lower (*p* < 0.05) in the XCB pigs at 185 D than in the pigs at 125 D.

**Table 8.** Differences in serum Ca and P concentrations (mmol/L) among different breeds of growing-finishing pigs.

Item	Duroc pig	XCB pig	TYB pig	SEM	*P*-values
Ca					
80 D	3.40^B^	3.31	3.42	0.12	0.928
125 D	3.86^A^	3.61	3.62	0.09	0.491
185 D	3.69^AB^	3.32	3.37	0.10	0.254
SEM	0.08	0.11	0.13		
*P*-values	0.040	0.437	0.730		
P					
80 D	4.08^a^	3.45^ABab^	2.99^Bb^	0.17	0.032
125 D	4.57	4.13^A^	4.05^A^	0.16	0.354
185 D	4.15^a^	3.13^Bb^	3.69^Aab^	0.13	0.002
SEM	0.12	0.17	0.17		
*P*-values	0.199	0.042	0.033		

Data are presented as means, SEM, and *P*-values. The replicates at 80, 125, and 185 D were 10 pigs per breed. ^a,b^The mean values with different small superscripts in the same row indicate significant differences among different pig breeds (*p* < 0.05). ^A,B^The mean values with different capital superscripts in the same column indicate significant differences among different day-old (*p* < 0.05). XCB, Xiangcun black; TYB, Taoyuan black; 80 D, 80 day-old; 125 D, 125 day-old; 185 D, 185 day-old.

### 3.8. Differences in bone Ca and P contents among three breeds of growing-finishing pigs

**Figure 3.**
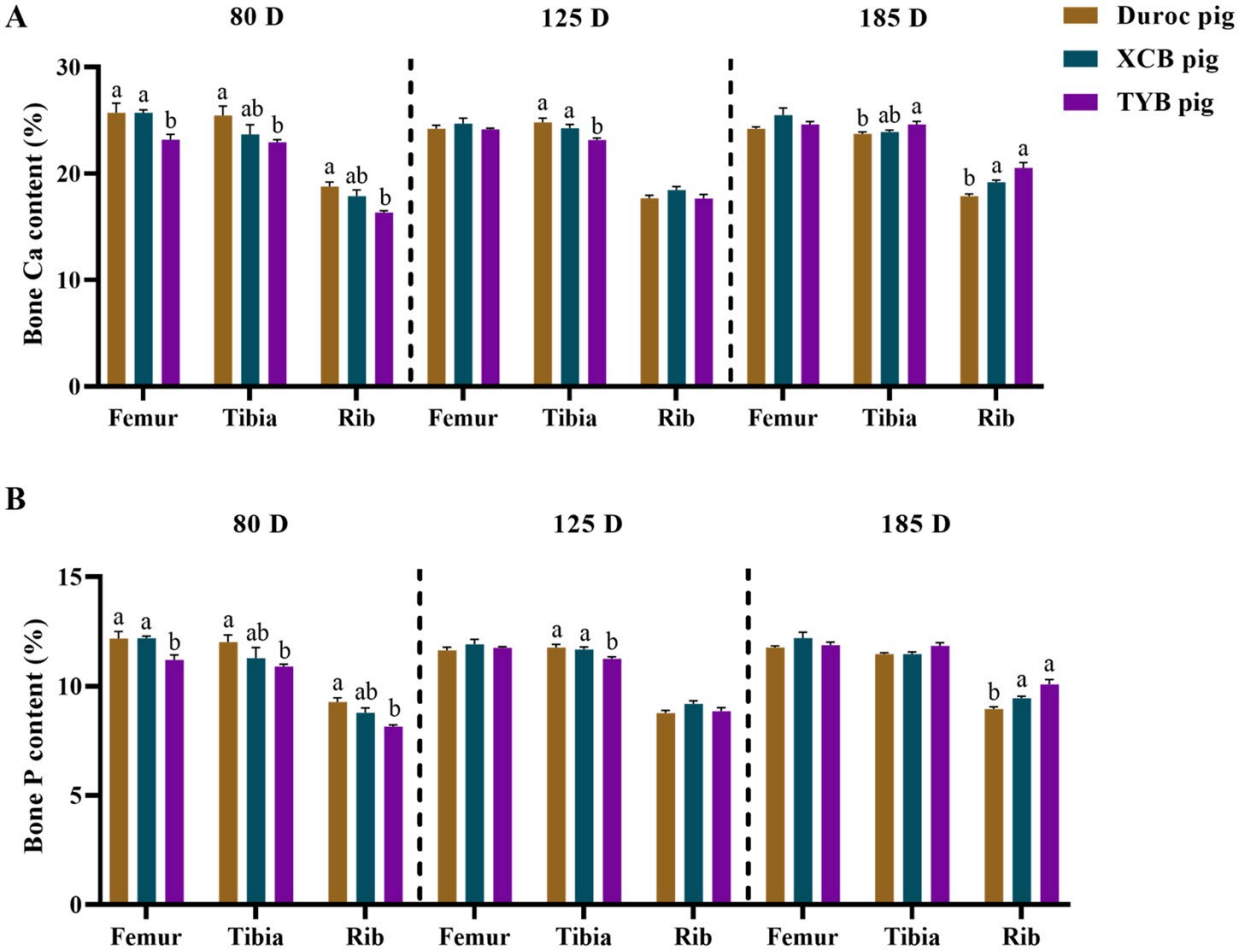
Differences in bone Ca (A) and P (B) contents among three breeds of growing-finishing pigs. The replicates at 80, 125, and 185 D were six per breed. Different small letters (a and b) indicate significant differences among different pig breeds at the same day-old (p < 0.05). 80, 125, and 185 D, represent 80, 125, and 185 day-old, respectively.

Compared with the Duroc and XCB pigs, the Ca and P contents were lower (*p* < 0.05) in the femur of TYB pigs at 80 D, as well as the Ca and P contents in the tibia at 125 D (Figure 3). Compared with the Duroc pigs, Ca and P contents in the tibia and rib of TYB pigs were lower (*p* < 0.05) at 80 D, while the tibia Ca content and the rib Ca and P contents of TYB pigs were higher (*p* < 0.05) at 185 D (Figure 3A–B).

### 3.9. Differences in serum biochemical indicators related to Ca and P metabolism among three breeds of growing-finishing pigs

**Figure 4.**
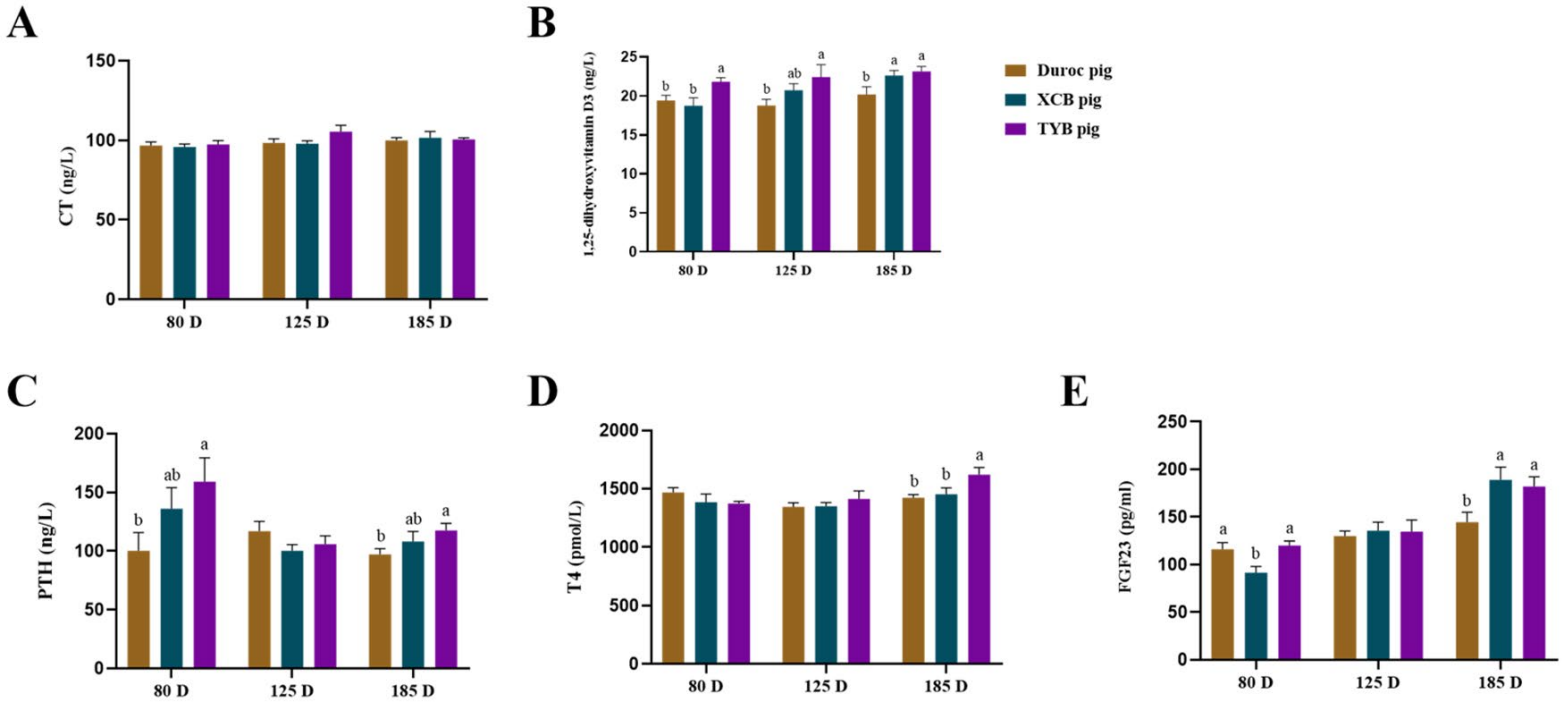
Differences in serum indicators related to Ca (A–C) and P (D–E) metabolism among three breeds of growing-finishing pigs. Different small letters (a and b) indicate significant differences among different pig breeds at the same day-old (p < 0.05). The replicates of duroc, XCB, and TYB pigs at 80 D were 9, 9, and 10, respectively. The replicates of duroc, XCB, and TYB pigs at 125 D were 9, 9, and 8, respectively. The replicates of duroc, XCB, and TYB pigs at 185 D were 9, 10, and 9, respectively. 80, 125, and 185 D represent 80, 125, and 185 day-old, respectively. CT = calcitonin; PTH = parathyroid hormone; T4 = thyroxine; FGF23 = fibroblast growth factor 23.

Serum 1,25-dihydroxyvitamin D_3_ concentration at 80, 125, and 185 D, PTH concentration at 80 D and 185 D, and T4 concentration at 185 D were higher (*p* < 0.05) in the TYB pigs than the Duroc pigs (Figure 4). Serum 1,25-dihydroxyvitamin D_3_ concentration of XCB pigs was higher (*p* < 0.05) at 185 D than the Duroc pigs. Compared with the XCB pigs, serum 1,25-dihydroxyvitamin D_3_ concentration at 80 D and T4 concentration at 185 D were higher (*p* < 0.05) in the TYB pigs (Figure 4A–C). As shown in Figure 4D–E, serum FGF23 concentration of XCB and TYB pigs was higher (*p* < 0.05) at 185 D than the Duroc pigs. Serum FGF23 concentration of Duroc and TYB pigs was higher (*p* < 0.05) at 80 D than the XCB pigs.

### 3.10. Differences in the mRNA expression of Ca and P transporter genes among three breeds of growing-finishing pigs

**Figure 5.**
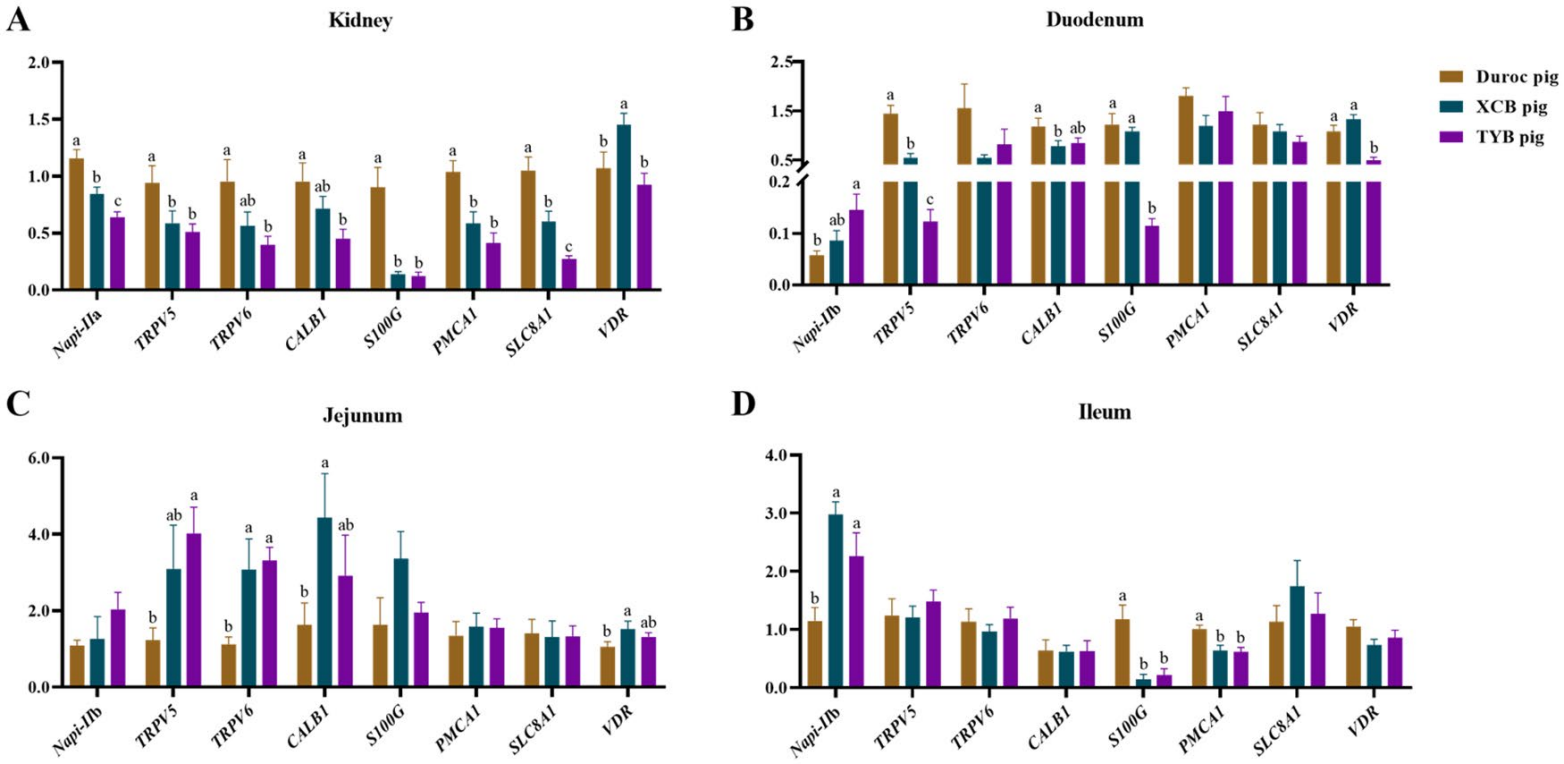
Differences in mRNA expressions of Ca and P transporters in the kidney (a), duodenum (B), jejunum (C), and ileum (D) of three different breeds of pigs at 80 day-old (D). The replicates were eight pigs per breed. Different small letters (a, b and c) indicate significant differences among different pig breeds (p < 0.05). *Napi-IIa* = sodium-dependent phosphate transport protein 2a; *Napi-IIb* = sodium-dependent phosphate transport protein 2b; *S100G* = S100 calcium binding protein G; *PMCA1* = plasma membrane calcium-transporting ATPase 1; *SLC8A1* = solute carrier family 8 member 1; *TRPV5* = transient receptor potential cation channel, subfamily V, member 5; *TRPV6* = transient receptor potential cation channel, subfamily V, member 6; *CALB1* = calbindin 1; *VDR* = vitamin D receptor.

The renal expressions of sodium-dependent phosphate transport protein 2a (*Napi-IIa*), plasma membrane calcium-transporting ATPase 1 (*PMCA1*), solute carrier family 8 member 1 (*SLC8A1*), transient receptor potential cation channel, subfamily V, member 5 (*TRPV5*), and S100 calcium binding protein G (*S100G*) of XCB and TYB pigs were downregulated (*p* < 0.05) at 80 D than the Duroc pigs (Figure 5). The renal expressions of transient receptor potential cation channel, subfamily V, member 6 (*TRPV6*) and calbindin 1 (*CALB1*) of TYB pigs were downregulated (*p* < 0.05) at 80 D versus the Duroc pigs. Furthermore, the renal vitamin D receptor (*VDR*) expression of Duroc and TYB pigs was downregulated (*p* < 0.05) compared to the XCB pigs (Figure 5A).

As shown in Figure 5B–D, the expressions of *Napi-IIb* in the duodenum and *TRPV5* in the jejunum were upregulated in the TYB pigs, while *CALB1* was downregulated in the duodenum of XCB pigs than the Duroc pigs (*p* < 0.05). The expressions of *CALB1* and *VDR* in the jejunum were upregulated in the XCB pigs, while *TRPV5* in the duodenum and *PMCA1* and *S100G* in the ileum were downregulated in the XCB and TYB pigs with respect to the Duroc pigs (*p* < 0.05). Moreover, the expressions of *TRPV6* in the jejunum and *Napi-IIb* in the ileum were upregulated (*p* < 0.05) in the XCB and TYB pigs than the Duroc pigs. Compared to the XCB pigs, the *TRPV5* expression in the duodenum was downregulated (*p* < 0.05) in the TYB pigs. The *S100G* and *VDR* expressions in the duodenum of Duroc and XCB pigs were upregulated (*p* < 0.05) in comparison to the TYB pigs.

**Figure 6.**
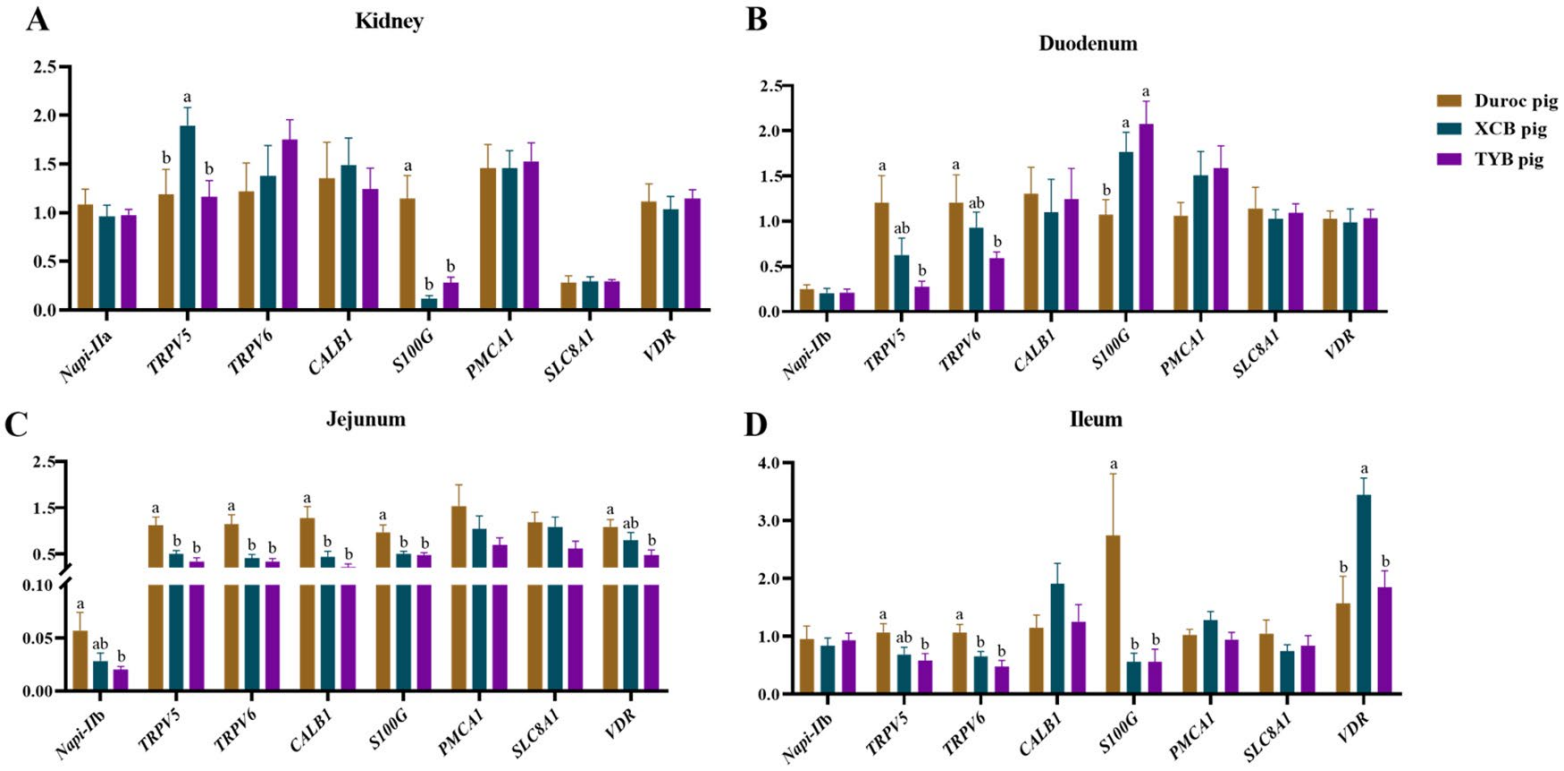
Differences in mRNA expressions of Ca and P transporters in the kidney (a), duodenum (B), jejunum (C), and ileum (D) of three different breeds of pigs at 125 day-old (D). The replicates were eight pigs per breed. Different small letters (a and b) indicate significant differences among different pig breeds (p < 0.05). *Napi-IIa* = sodium-dependent phosphate transport protein 2a; *Napi-IIb* = sodium-dependent phosphate transport protein 2b; *S100G* = S100 calcium binding protein G; *PMCA1* = plasma membrane calcium-transporting ATPase 1; *SLC8A1* = solute carrier family 8 member 1; *TRPV5* = transient receptor potential cation channel, subfamily V, member 5; *TRPV6* = transient receptor potential cation channel, subfamily V, member 6; *CALB1* = calbindin 1; *VDR* = vitamin D receptor.

The renal *S100G* expression of XCB and TYB pigs was upregulated (*p* < 0.05) in relation to the Duroc pigs (Figure 6). The renal *TRPV5* expression of Duroc and TYB pigs was downregulated (*p* < 0.05) in contrast to the XCB pigs (Figure 6A). As shown in Figure 6B–D, the expressions of *TRPV5*, *TRPV6*, *CALB1*, and *S100G* in the duodenum, *TRPV6* in the ileum, and *S100G* in the jejunum of XCB and TYB pigs were downregulated (*p* < 0.05) than the Duroc pigs. The expressions of *TRPV5* and *TRPV6* in the duodenum, *Napi-IIb* and *VDR* in the jejunum, and *TRPV5* in the ileum were downregulated (*p* < 0.05) in the TYB pigs, while *S100G* was upregulated (*p* < 0.05) in the duodenum of XCB and TYB pigs more than the Duroc pigs. Moreover, the *VDR* expression in the ileum of Duroc and TYB pigs was downregulated (*p* < 0.05) compared to the XCB pigs.

**Figure 7.**
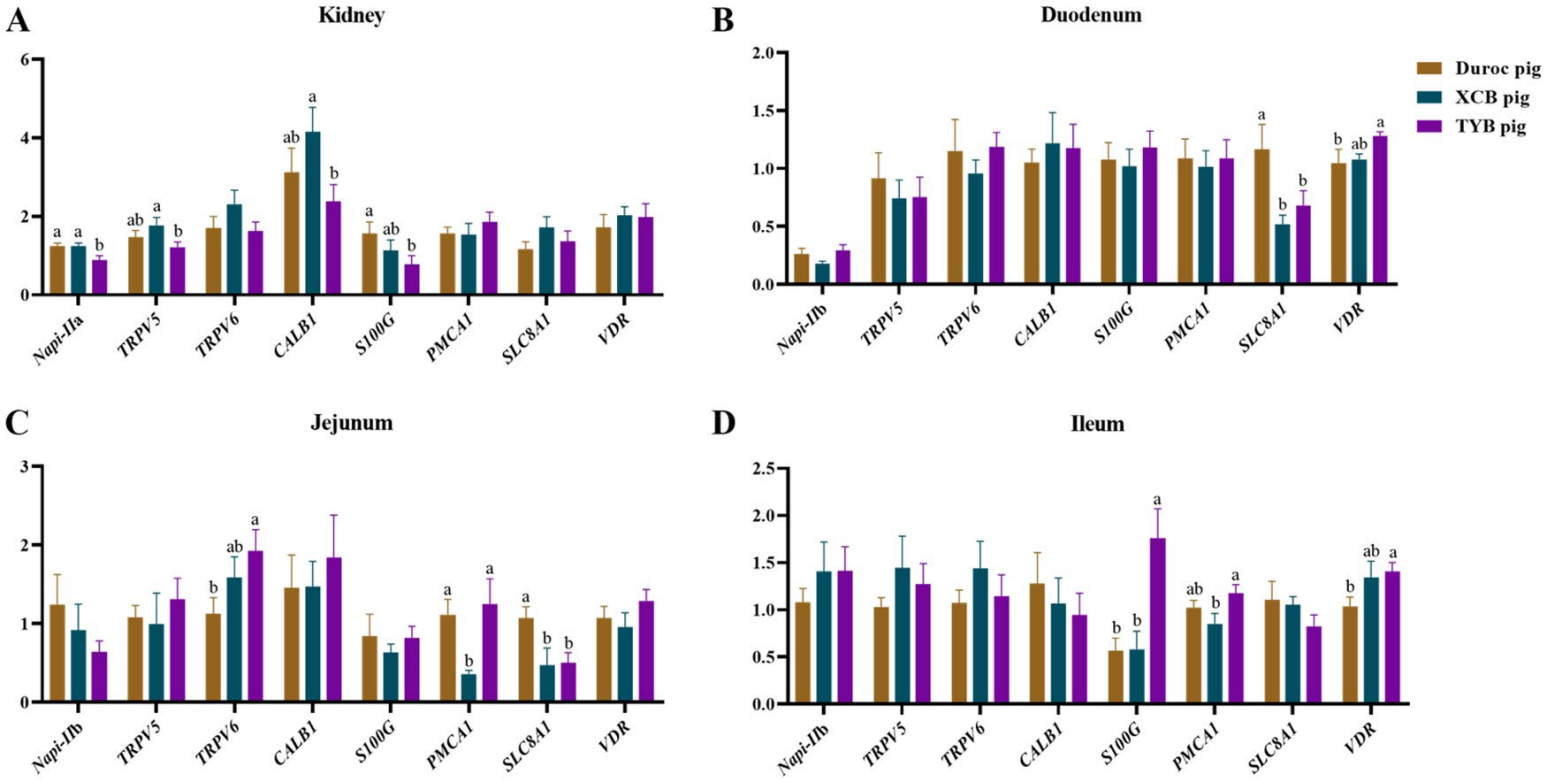
Differences in mRNA expressions of Ca and P transporters in the kidney (a), duodenum (B), jejunum (C), and ileum (D) of three different breeds of pigs at 185 day-old (D). The replicates were eight pigs per breed. Different small letters (a and b) indicate significant differences among different pig breeds. *Napi-IIa* = sodium-dependent phosphate transport protein 2a; *Napi-IIb* = sodium-dependent phosphate transport protein 2b; *S100G* = S100 calcium binding protein G; *PMCA1* = plasma membrane calcium-transporting ATPase 1; *SLC8A1* = solute carrier family 8 member 1; *TRPV5* = transient receptor potential cation channel, subfamily V, member 5; *TRPV6* = transient receptor potential cation channel, subfamily V, member 6; *CALB1* = calbindin 1; *VDR* = vitamin D receptor.

Compared to Duroc pigs, the renal *S100G* expression was downregulated (*p* < 0.05) in the TYB pigs (Figure 7). The expression of renal *Napi-IIa* was upregulated (*p* < 0.05) in the Duroc and XCB pigs, as well as the renal *TRPV5* and *CALB1* in the XCB pigs compared to the TYB pigs (Figure 7A). The expressions of *SLC8A1* in the duodenum and *SLC8A1* in the jejunum of XCB and TYB pigs were downregulated, while *VDR* in the duodenum and ileum and *TRPV6* in the jejunum of TYB pigs were upregulated compared to the Duroc pigs (*p* < 0.05). Compared to the XCB pigs, the expression of *PMCA1* was upregulated in the jejunum of Duroc and TYB pigs, as well as the *PMCA1* in the ileum of TYB pigs (*p* < 0.05). The *S100G* expression in the ileum of Duroc and XCB pigs was downregulated (*p* < 0.05) than in the TYB pigs (Figure 7B–D).

## 4. Discussion

Bone is conventionally considered as a structural organ involved in the secretion of cytokines and the protection and mechanical support of the body. The physiological bone modeling (including bone formation and absorption) occurs constantly throughout life and maintains bone function, integrity, and mineral homeostasis (Raggatt and Partridge 2010; Han et al. 2018). Bone characteristics are significantly affected by Ca, P, and protein levels in diets of commercial pigs (Chen et al. 2002; Liesegang et al. 2002; Weremko et al. 2013); however, these profiles in Chinese domestic pigs still need to be elucidated. Thus, the present study comprehensively assessed the skeletal conditions and differences in gene expression related to renal uptake of Ca, P, and bone metabolism in four functional bones of different breeds of growing-finishing pigs. The results showed that the bone characteristics are significantly different among different pig breeds, which may be associated with the changes in serum biochemical markers, as well as renal and small intestinal mRNA expressions related to Ca and P metabolism.

Commercial pig breeds (especially Duroc pigs) have a higher growth rate than Chinese domestic pigs (Young 1998), while the inclusion of Chinese domestic pig breeds in hybrid leads to lighter bones (Cesar et al. 2010). The present study showed that the length and weight of the femur, tibia, rib, and lumbar vertebrae were lower in the XCB and TYB pigs at different age stages. These inconsistencies might be due to the genetically smaller body size of Chinese domestic pigs than that of the Duroc pigs at the same age stage. The bone index could assess skeletal growth. Although the bone index of the femur, tibia, and rib did not differ among different pig breeds at the early age (80 D), those indexes were higher in the Duroc pigs at 185 D, suggesting that Duroc pigs have a relatively higher skeletal growth rate during the late-finishing period. Further in-depth studies related to skeletal growth rate among different breeds of pigs warrant to reveal the exact mechanism.

The BMC reflects the mineral deposition of the bone scanned, whose value is also related to the bone size. Although there was no significant difference in the rib mineral content between the Duroc pigs and TYB pigs at 185 D, the weight and length of the rib of TYB pigs were significantly lower and smaller in the present study. Previous studies reported that bone mineral deposition in growing pigs does not depend on the body protein, whereas it depends on the BW in terms of potential and actual mineral deposition (Lautrou et al. 2020). Therefore, we postulated that within the same age, as the BW of TYB pigs is lower which might have higher mineral deposition capacity with the fact that TYB pigs had lower weight and shorter rib lengths.

The BMD is a quantitative indicator of osteopenia and osteoporosis (Ferretti et al. 2003), as well as a physiological parameter for bone health evaluation. Our findings showed that Duroc pigs have higher BMD in the femur and tibia compared with the XCB and TYB pigs at 80 D and 125 D, indicating a better development of posterior limb bone in Duroc pigs. BMD increases at the early growth stage to adapt to the explosive increase of external loads, while trabecular structural optimization occurs after 161 D to further enhance bone mechanical properties (Tanck et al. 2001). The bone mass deposition replacement with skeletal structural rearrangement during the late finishing period did not affect the BMD among the different pig breeds at 185 D in the present study.

Bone breaking load is a representative indicator of the maximum load capacity of bone (Töyräs et al. 1999). Duroc pigs presented higher bone breaking load of the femur and tibia during the finishing stage, but TYB pigs had higher bone breaking load at 185 D, which is consistent with the BMC value. These findings indicate that the TYB pigs have a higher potential for rib development during the late-finishing stage. We also found that TYB pigs have lower bone ash content compared with the Duroc and XCB pigs at 80 D, while it was higher in the tibia and rib at 125 D and 185 D, suggesting that the TYB pigs experienced a higher level of bone mineralization during the finishing stage. A previous study also reported that pigs fed with Ca restricted diet had a lower bone ash content but had no significant difference in BMC (Bai et al. 2017), indicating that bone ash content can be more sensitive than BMC. In addition, the ash content of femurs of different breeds of pigs was consistent throughout the trial, while the ash content in the tibia and rib fluctuated. According to Lee et al. (2021), the tibia can be more representative for evaluating the total bone mineralization than the femur and rib. Collectively, TYB pigs presented slower mineral deposition in relation to the Duroc and XCB pigs at the beginning of the finishing stage, but had a higher potential for bone mineralization from 80 D to 185 D. Therefore, standardization of mineral contents in diets at the beginning of the finishing stage of TYB pigs will have a great significance to improve mineral deposition in TYB pigs.

The bone weight, length, strength, mineral content, and density were higher from 80 D to 185 D regardless of the breed, indicating stronger bone development as age increases. In addition, the bone index decreased in the three breeds of pigs from 80 D to 125 D. The bone weight increases relatively slower than the BW in growing-finishing pigs (Richmond and Berg 1972). However, the bone index did not differ among XCB and TYB pigs from 120 D to 185 D, while it was increased in the Duroc pigs. Genetic factors might be more prone to associate with the metabolic activity of the bones in Duroc pigs than the other two domestic pig breeds; however, further studies warrant revealing the exact mechanism. As the bone index represents the bone mass required per kg of BW, thus, Chinese domestic pigs may allocate more weight to muscle and fat instead of bone compared to the Duroc pigs.

Serum biomarkers are the indicators of bone turnover and bone cell activities (Allen 2003). The TYB pigs presented higher serum BMP-2 and OC levels than the Duroc pigs at 125 D and 185 D in the present study. Previously, it has been found that serum OC level is negatively correlated to the age of pigs and bone ash content (Carter et al. 1996). These findings suggest that the bone formation of TYB pigs occurs at the finishing stage, which is consistent with the increased ash content observed in the present study. Serum TRACP5b secreted by osteoclasts is a bone resorption marker (Henriksen et al. 2007). Our findings showed that serum BALP concentration had an opposite trend with other markers, whereas TYB pigs had lower serum TRACP5b concentration compared with the Duroc and XCB pigs at 125 D, indicating that the bone turnover was affected by bone formation rather than absorption.

We also observed that serum CTX-1 concentration was higher and TRACP5b concentration was lower in the TYB pigs at 80 D. The CTX-1 is known as a marker of bone degradation, as well as an independent predictor of early femur loss (Dolgos et al. 2009). Collagen matrix content reduction may lead to bone weakness (Silva et al. 2006), while no evidence was found for bone weakness of TYB pigs in the present study. Therefore, the bone formation of TYB pigs was higher at 125 D and 185 D, while Duroc and XCB pigs had better skeletal development at the finishing stage.

The ATTD of Ca and P is calculated by the difference between mineral intake and fecal excretion, while the available minerals for bone formation can be evaluated by other methods, such as bone mineralization (Salguero et al. 2014). In the present study, Ca and P contents of the femur, tibia, and rib of TYB pigs were lower compared to Duroc pigs at 80 D. Mineralization in the femur and rib showed a response to the Ca and P contents of pigs at the beginning of the growing-finishing stage. These results suggest a lower mineralization level of TYB pigs at 80 D, which is consistent with the decreased ash content in the femur, tibia, and ribs of TYB pigs compared with the Duroc and XCB pigs in the present study. Despite a higher ATTD of Ca and P in the TYB pigs, nutrient digestibility includes apparent and true digestibility, and a higher endogenous losses may be resulted in lower Ca and P contents in the femurs. Furthermore, compared with the Duroc pigs, Ca and P contents were higher in the rib of TYB pigs at 185 D, suggesting a higher Ca and P deposition at the late finishing stage.

The vitamin D hormone (1,25-dihydoxyvitamin D_3_) is one most important factors to increase the intestinal Ca absorption (Silva et al. 2006). In the present study, serum 1,25-dihydroxyvitamin D_3_ level was higher in the TYB pigs at 80, 125, and 185 D compared with the Duroc pigs, suggesting that TYB pigs have a higher Ca absorption at the growing-finishing stage. In addition, PTH and CT regulate the activities of osteoblasts and osteoclasts, respectively, to maintain Ca homeostasis (Carter and Schipani 2006). In the present study, serum T4 and FGF23 levels of TYB pigs were higher at 185 D compared with the Duroc pigs. FGF23 can reduce the serum P level by inhibiting renal reabsorption (Shimada et al. 2004) and then combine with T4 to maintain the P homeostasis. The increased P uptake in the intestine may reduce renal reabsorption reduction to keep a normal serum P level.

The *Napi-IIa* is a P metabolism-related gene responsible for approximately 85% of total renal P uptake (Murer et al. 2003). In the present study, compared with the Duroc pigs, renal *Napi-IIa* expression of TYB pigs was lower at 80 D, while a higher *Napi-IIb* expression was also observed. Higher intestinal uptake may occur as compensation for the lower renal uptake level of P in the TYB pigs at 80 D.

The ion channels *TRPV5* and *TRPV6* transport Ca from the lumen to the absorbing enterocyte, while the intracellular calbindins, including *S100G* and *CALB1*, accelerate Ca trans-cytosolic diffusion. The ATP-activated Ca pump *SLC8A1* and *PMCA1* are responsible for the discharge of Ca in the enterocyte (Wasserman 2004; van de Graaf et al. 2007). In the present study, Ca uptake-related gene expressions were higher in the duodenum and ileum of Duroc pigs at 80 D among the three pig breeds, while the jejunal ones were lower at the same age. Meanwhile, Duroc pigs had higher renal Ca uptake-related gene expression. Although the activity of Ca transporter in the duodenum is higher than in the jejunum and ileum, mostly intestinal Ca uptake occurs in the ileum because of the relatively longer transition time (Duflos et al. 1995). These findings suggest that both intestinal and renal Ca uptake levels in the Duroc pigs are higher in comparison to the Chinese domestic pigs at early growing-finishing stage. In addition, we observed higher small intestinal *TRPV5* and *TRPV6* expressions in the Duroc pigs at 125 D, indicating a stronger Ca transportation ability in the enterocyte from the gut lumen. Moreover, the *SLC8A1* expression in the duodenum and jejunum was lower, while the *TRPV6* in the jejunum and *S100G* in the ileum were higher in the TYB pigs compared to the Duroc pigs at 185 D. These findings suggest that Ca uptake was enhanced in the TYB pigs with the absence of significant differences in Ca excretion at 185 D. The 1,25-dehroxyvitamin D_3_ regulates both renal and intestinal Ca uptake proteins *via VDR* expressed in the renal proximal or distal tubule and small intestine (Lambers et al. 2006; Maiti and Beckman 2007). Our findings showed that the *VDR* expression in the duodenum and ileum was higher in the TYB pigs compared with the Duroc pigs at 185 D, as well as the serum 1,25-dihydroxyvitamin D_3_ concentration, suggesting a superiority of 1,25-dihydorxyvitamin D_3_ mediated intestinal Ca absorption in the TYB pigs.

## 5. Conclusion

In summary, bone parameters and gene expression related to Ca and P uptake differed among different breeds of pigs. The TYB pigs had better bone development indicated by higher serum bone formation (BMP-2, OC, and PINP) and lower bone resorption (TRACP5b) at 125 D. The mineral content and bone breaking load of the rib were relatively higher in the TYB, as well as the Ca and P contents, which may result from a higher mineral deposition. Duroc pigs have a more active Ca transportation than Chinese domestic pigs at the early finishing stage by upregulating Ca-transporter related gene expressions, whereas TYB pigs have higher Ca absorption capacity through increasing the serum PTH and 1,25-dihydroxyvitamin D_3_ concentrations and upregulating *S100G* and *VDR* expressions at the finishing stage. Collectively, TYB pigs showed a higher potential for Ca utilization at the finishing stage. These findings have great implications to modify or improving the Ca and P contents in diets of domestic pigs. Our findings also provide guiding evidence for bone development and mineral uptake among different pig breeds and provide basic data for breeding and preserving Chinese domestic pigs. Moreover, further in-depth studies are necessary to reveal the potential of mineral composition in diets on bone behavior and health outcomes, as well as mechanistic insights into how genetic variations contribute to bone characteristics.

## Supplementary Material

Supplemental Material

## Data Availability

The original contributions presented in the study are included in the article [and/or] its supplementary materials.
