# Supplementary material for: Differences in intestinal and renal Ca and P uptake in three different breeds of growing-finishing pigs
Source: Vet Q. 2024 Jul 4;44(1):1–16. doi: 10.1080/01652176.2024.2371609 (PMC11229737; doi:10.1080/01652176.2024.2371609)
Supplement: Supplemental Material [file TVEQ_A_2371609_SM0952.docx]

**Supplementary Materials**

**Table S1**

Ingredients and nutritional levels of diets, % as dry matter basis.

| Item | Pre-nursey period  (8−20 kg) | Late-nursey period  (20−35 kg) | Growing period  (35−60 kg) | Finishing period  (60−90 kg) |
| --- | --- | --- | --- | --- |
| Ingredients |  |  |  |  |
| Corn (8% CP) | 36.42 | 63.08 | 69.59 | 64.73 |
| Extruded corn (7.8% CP) | 27.00 | — | — | — |
| Extruded soybean (35.5% CP) | 3.00 | 6.00 | — | — |
| Soybean meal (44% CP) | 19.80 | 16.59 | 17.14 | 12.50 |
| Fish meal (62.5% CP) | 5.97 | 1.80 | 2.30 | — |
| Whey powder (low protein) | 4.50 | — | — | — |
| Wheat bran (14.3% CP) | — | 9.37 | 9.00 | 20.45 |
| Soybean oil | 1.00 | 0.50 | — | — |
| Limestone powder | 0.70 | 0.90 | 0.65 | 0.80 |
| CaHPO_4_ | 0.40 | 0.65 | 0.35 | 0.30 |
| NaCl | 0.30 | 0.30 | 0.30 | 0.30 |
| L-Lysine hydrochloride (78%) | 0.30 | 0.20 | 0.15 | 0.40 |
| DL-Methionine | 0.08 | 0.03 | 0.03 | 0.02 |
| L-Threonine (98.5%) | 0.10 | 0.17 | 0.09 | 0.10 |
| L-Tryptophan (98%) | 0.04 | 0.02 | 0.01 | 0.01 |
| Choline chloride | 0.15 | 0.15 | 0.15 | 0.15 |
| Vitamin premix^1^ | 0.04 | 0.04 | 0.04 | 0.04 |
| Mineral premix^2^ | 0.20 | 0.20 | 0.20 | 0.20 |
| Total | 100.00 | 100.00 | 100.00 | 100.00 |
| Nutrient levels^3^ |  |  |  |  |
| Digestible energy (MJ/kg) | 14.19 | 13.73 | 13.48 | 12.98 |
| Crude protein | 19.11 | 17.27 | 16.05 | 14.04 |
| Calcium | 0.81 | 0.76 | 0.58 | 0.53 |
| Available phosphorus | 0.42 | 0.34 | 0.28 | 0.22 |
| Lysine | 1.22 | 0.93 | 0.81 | 0.84 |
| Methionine | 0.39 | 0.28 | 0.26 | 0.20 |
| Methionine + cysteine | 0.66 | 0.55 | 0.52 | 0.44 |
| Threonine | 0.76 | 0.70 | 0.56 | 0.49 |
| Tryptophan | 0.24 | 0.19 | 0.17 | 0.15 |

^1^ Providing the following amounts of vitamins per kilogram of a complete diet: 1,800 IU vitamin A, 200 IU vitamin D_3_, 11 mg vitamin E, 0.5 mg vitamin K, 1 mg vitamin B_1_, 3.5 mg vitamin B_2_, 1.5 mg vitamin B_6_, 17.5 mg vitamin B_12_, 15 mg niacin, 10 mg pantothenic acid, 0.3 mg folate, and 0.05 mg biotin during 8−20 kg BW; 5,000 IU vitamin A, 20 mg vitamin C, 50 mg vitamin E, 1 mg vitamin K, 2 mg vitamin B_1_, 10 mg vitamin B_2_, 4 mg vitamin B_6_, 0.03 mg vitamin B_12_, 30 mg niacin, 20 mg pantothenic acid, 0.6 mg folate, and 0.3 mg biotin during 20−35 kg BW; and 2,250 IU vitamin A, 220 IU vitamin D_3_, 16 mg vitamin E, 0.5 mg vitamin K, 2 mg vitamin B_1_, 5 mg vitamin B_2_, 4 mg vitamin B_6_, 0.03 mg vitamin B_12_, 30 mg niacin, 20 mg pantothenic acid, 0.3 mg folate, and 0.2 mg biotin during 35−60 and 60−90 kg BW.

^2^ Providing the following amounts of minerals per kilogram of a complete diet: 105 mg Fe, 6 mg Cu, 100 mg Zn, 4 mg Mn, 0.14 mg I, and 0.3 mg Se during 8−20 kg BW; 180 mg Fe, 12 mg Cu, 150 mg Zn, 3 mg Mn, 0.14 mg I, and 0.25 mg Se during 20−35 kg BW; 100 mg Fe, 10 mg Cu, 100 mg Zn, 2 mg Mn, 0.14 mg I, and 0.25 mg Se during 35−60 kg BW; and 80 mg Fe, 5 mg Cu, 80 mg Zn, 3 mg Mn, 0,14 mg I, and 0.25 mg Se during 60−90 kg BW.

^3^ Data are the results of chemical analysis conducted in triplicate.

**Table S2**

Specific primers sequences of the target genes for this study.

| Gene names | Accession No. | Primer sequences (5′−3′) | Product size (bp) |
| --- | --- | --- | --- |
| *CALB1* | NM_001130226.1 | F: ATTTCGACGCTGACGGAAGT | 224 |
|  |  | R: TTGCTGGCATCGGAATAGCA |  |
| *NaPi-IIa* | NM_001044623.1 | F: ACAGAACACCAAGGGCCCAC | 130 |
|  |  | R: CACGTAGGCAAAGGTCGCC |  |
| *NaPi-IIb* | NM_001256772.1 | F: GCCACTGTCCACGACTTCTT | 132 |
|  |  | R: GGCATCCTCTCCACTCTTGA |  |
| *PMCA1* | NM_214352.3 | F: GCCGGGCGAGATGTGTA | 269 |
|  |  | R: CTCCACCATAGGCAACCGAG |  |
| *SLC8A1* | XM_021088307.1 | F: GAAGGACAAGCATCCCAGGTC | 209 |
|  |  | R: CCCTTCACTGATGGCAAGGTT |  |
| *S100G* | NM_214140.2 | F: CAGGACACCAAAATGAGTGCC | 164 |
|  |  | R: TCTAGGGTTCTCGGACCTTTCA |  |
| *TRPV5* | XM_021078896.1 | F: CCCTTGAACCACATCCCTGT | 193 |
|  |  | R: GAGGTGACTCTCGAATCCTCTTC |  |
| *TRPV6* | XM_021078898.1 | F: CCAATCACGAGGGTCTCACC | 259 |
|  |  | R: CTCCACTTGAGGCTCACCAG |  |
| *VDR* | NM_001097414.1 | F: GTTGGAAGTGTCTGGGAGCC | 237 |
|  |  | R: CGCTTCATGCTCCGTCTGAA |  |
| β-actin | XM_021086047.1 | F: GGCACCACACCTTCTACAACGAG | 102 |
|  |  | R: TCATCTTCTCACGGTTGGCTTTGG |  |

*CALB1* = calbindin 1; *Napi-IIa* = Sodium-dependent phosphate transport protein 2a; *Napi-IIb* = Sodium-dependent phosphate transport protein 2b; *S100G* = S100 calcium binding protein G; *PMCA1* = plasma membrane calcium ATPase 1; *SLC8A1* = solute carrier family 8 member 1; *TRPV5* = transient receptor potential cation channel, subfamily V, member 5; *TRPV6* = transient receptor potential cation channel, subfamily V, member 6; *VDR* = vitamin D receptor.

**Table S3**

Differences in growth performance among three breeds of growing-finishing pigs.

| Item | Duroc pig | XCB pig | TYB pig | SEM | *P*-values |
| --- | --- | --- | --- | --- | --- |
| **35−80 D** |  |  |  |  |  |
| Initial BW (kg) | 6.68^b^ | 5.63^b^ | 10.18^a^ | 0.48 | <0.001 |
| ADG (kg/day) | 0.11^b^ | 0.10^b^ | 0.44^a^ | 0.03 | <0.001 |
| ADFI (kg/day) | 0.26^b^ | 0.17^c^ | 1.03^a^ | 0.08 | <0.001 |
| FCR | 2.53^a^ | 1.61^b^ | 2.40^a^ | 0.12 | 0.002 |
| Final BW (kg) | 11.38^b^ | 9.42^b^ | 22.94^a^ | 1.26 | <0.001 |
| **80−125 D** |  |  |  |  |  |
| Initial BW (kg) | 10.46^b^ | 10.66^b^ | 24.93^a^ | 1.42 | <0.001 |
| ADG (kg/day) | 0.54^b^ | 0.50^b^ | 0.81^a^ | 0.03 | <0.001 |
| ADFI (kg/day) | 1.21^b^ | 1.15^b^ | 1.89^b^ | 0.08 | <0.001 |
| FCR | 2.24 | 2.33 | 2.36 | 0.05 | 0.626 |
| Final BW (kg) | 34.79^b^ | 33.23^b^ | 61.33^a^ | 2.81 | <0.001 |
| **125−185 D** |  |  |  |  |  |
| Initial BW (kg) | 37.92^b^ | 36.77^b^ | 58.47^a^ | 2.13 | <0.001 |
| ADG (kg/day) | 0.66^b^ | 060^b^ | 0.77^a^ | 0.02 | <0.001 |
| ADFI (kg/day) | 2.62^b^ | 2.60^b^ | 3.46^a^ | 0.09 | <0.001 |
| FCR | 4.10 | 4.40 | 4.52 | 0.10 | 0.262 |
| Final BW (kg) | 77.08^b^ | 72.60^b^ | 104.65^a^ | 3.03 | <0.001 |

Data are presented as means, SEM, and *P*-values. The replicates at 35−80 D, 80−125 D, and 125−185 D were 10 pigs per breed. ^a−c^ The mean values with different small superscripts in the same row indicate significant differences among different pig breeds (*P* < 0.05). XCB, Xiangcun black; TYB, Taoyuan black; 80 D, 80 day-old; 125 D, 125 day-old; 185 D, 185 day-old.
